# Population structure of honey bees in the Carpathian Basin (Hungary) confirms introgression from surrounding subspecies

**DOI:** 10.1002/ece3.1781

**Published:** 2015-11-04

**Authors:** Erika Péntek‐Zakar, Andrzej Oleksa, Tomasz Borowik, Szilvia Kusza

**Affiliations:** ^1^ Institute of Animal ScienceBiotechnology and Nature ConservationUniversity of Debrecen4032DebrecenHungary; ^2^ Department of GeneticsKazimierz Wielki University85‐064BydgoszczPoland; ^3^ Mammal Research InstitutePolish Academy of Sciences17‐230BialowiezaPoland

**Keywords:** Carniolan honey bee, genetic diversity, Hungary, introgression, microsatellite, mitochondrial DNA

## Abstract

Carniolan honey bees (*Apis mellifera carnica*) are considered as an indigenous subspecies in Hungary adapted to most of the ecological and climatic conditions in this area. However, during the last decades Hungarian beekeepers have recognized morphological signs of the Italian honey bee (*Apis mellifera ligustica*). As the natural distribution of the honey bee subspecies can be affected by the importation of honey bee queens or by natural gene flow, we aimed at determining the genetic structure and characteristics of the local honey bee population using molecular markers. All together, 48 Hungarian and 84 foreign (Italian, Polish, Spanish, Liberian) pupae and/or workers were used for mitochondrial DNA analysis. Additionally, 53 sequences corresponding to 10 subspecies and the Buckfast hybrid were downloaded from GenBank. For the nuclear analysis, 236 Hungarian and 106 foreign honey bees were genotyped using nine microsatellites. Heterozygosity values, population‐specific alleles, FST values, principal coordinate analysis, assignment tests, structure analysis, and dendrograms were calculated. Haplotype and nucleotide diversity values showed moderate values. We found that one haplotype (H9) was dominant in Hungary. The presence of the black honey bee (*Apis mellifera mellifera*) was negligible, but a few individuals resembling other subspecies were identified. We proved that the Hungarian honey bee population is nearly homogeneous but also demonstrated introgression from the foreign subspecies. Both mitochondrial DNA and microsatellite analyses corroborated the observations of the beekeepers. Molecular analyses suggested that Carniolan honey bee in Hungary is slightly affected by Italian and black honey bee introgression. Genetic differences were detected between Polish and Hungarian Carniolan honey bee populations, suggesting the existence of at least two different gene pools within *A. m. carnica*.

## Introduction

Honey bees provide an important pollination services in commercial crops and in many natural habitats worldwide (Klein et al. 2007). Based on the estimates, approximately 35% of human food consumption depends directly or indirectly on insect‐mediated pollination (Delaplane and Mayer 2000).

The evolutionary history of the species *Apis mellifera* (Linnaeus, 1758) was first determined based on morphometric parameters (Ruttner et al. 1978). *A. mellifera* has up to 30 subspecies in different regions of the world (Ruttner 1988). These subspecies were classified into four main groups. One of them is the C lineage that includes north Mediterranean subspecies as *A. m. carnica*, and a second one named M contains northern and western European subspecies as *A. m. mellifera* and *A. m. iberiensis*, respectively. The two major lineages of honey bee in Europe arose from two independent migration events from source populations in Africa (Whitfield et al. 2006). The A group includes African subspecies, and the Oriental O group comprises subspecies mainly spread in the Middle East (Ruttner 1992) (see Fig. S1). With the advent of molecular techniques, these groups have been further confirmed (Wallberg et al. 2014). Furthermore, two new lineages have been added: the Y lineage in Ethiopia (Franck et al. 2001) and a recently described fifth independent nuclear cluster called Z containing those honey bee populations spread in Libya (Alburaki et al. 2013).

The honey bees of lineage (C) are variable in behavior and color and in addition adapted to various climatic zones from Mediterranean climate to colder mountains of the Balkans and Central Europe (Ruttner 1988). The Carniolan honey bee, *A. m. carnica* (Pollmann, 1979), is native to Hungary, Slovenia, and some regions of the former Yugoslavia, Romania, Bulgaria, and southern Austria (Ruttner 1988; Oleksa et al. 2013). Lately, due to wide human‐assisted dissemination of Carniolan queens, the subspecies has expanded from its native range to central and northern European countries and also to Canada, the United States, and other parts of the world (Ruttner 1992). Ruttner (1988) described local morphometric ecotypes according to zoogeographic zones (Alpine, Pannonian, and Dalmatian) within this subspecies, but in 1992, the same author concluded the existence of only Pannonian (Hungary, Croatia, Romania) and Alpine (Austria, Slovenia) ecotypes and several regional variations. An example of the existence of regional variations was demonstrated by Muñoz et al. (2009) through the molecular analyses of the honey bee population form Croatia.

The adaptation of honeybees to their local environment has not been well studied (Meixner et al. 2014). The Pannonian honey bee is endemic to the Carpathian Basin, which results from long‐term evolution, migration, and adaptation processes, which started long before human influences came into the area. Accordingly, there is special importance to maintain our diverse ecotype.

Mitochondrial DNA (mtDNA) analysis has become a widely used approach in studying the genetic diversity among populations because of its conserved gene content, high level of nucleotide substitutions, and maternal inheritance. The most widely used marker was the intergenic region between the cytochrome oxidase I and II (cox1–cox2) genes in *A. mellifera* mtDNA, which can be used to infer honey bee evolutionary relationships (Garnery et al. 1993; Stevanovic et al. 2010; Magnus et al. 2011, 2014; Yin and Ji 2013; Chalapathy et al. 2014). The five above‐mentioned evolutionary lineages of honey bees have also been depicted by studying the highly variable cox1–cox2 intergenic region (Cornuet et al. 1991) and confirmed that *A. m. carnica* belongs to the eastern Mediterranean mitochondrial linage (C linage). Five haplotypes were initially described within the C lineage: C1 in *A. m. ligustica*, C2a in *A. m. carnica*, C2b in *A. m. caucasica* (Franck et al. 2000), C2d in A*. m. macedonica*, and C2c in *A. m. carnica* in Slovenia and Croatia (Susnik et al. 2004). In addition, C2e was identified in *A. m. carnica* in Serbia (Kozmus et al. 2007) and Croatia (Muñoz et al. 2009) but the number of haplotypes is continuously increasing and up to 11 new haplotypes have been reported by Coroian et al. (2014) in honey bees from Romania.

Microsatellites are biparentally inherited markers and give useful information about population events such as introgression and hybridization through mating between foreign drones and local queens (Jensen et al. 2005). Microsatellite studies on honey bee populations have been generally carried out for European and African subspecies (Franck et al. 1998, 2001). In this sense, *A. m. mellifera* populations from Norway, Sweden, Denmark, England, Scotland, and Ireland were checked for introgression (Jensen et al. 2005) and the most introgressed population was found on the Danish Island of Laeso. According to Il'yasov et al. (2015), only four local black bee populations are kept as pure black bee in Russia. In the Mediterranean honey bee populations, microsatellite analysis revealed the presence of carnica‐characterizing alleles in the known natural hybrid zones and also in the north of the Veneto region in Italy (Dall'Olio et al. 2007) and on Sicily island, thus interfering with the conservation of the endemic subspecies *A. m. siciliana* (Muñoz et al. 2014). Oleksa et al. (2011) with microsatellites showed the presence of hybrids since from 10 to 30% of the nuclear genes in the black honey bee (*A. m. mellifera*) populations in Polish Augustów Forest derived from nonnative bees.

*A. m. carnica* and *A. m. ligustica* have been considerably imported by beekeepers (De la Rúa et al. 2009), therefore risking the conservation of native honey bee subspecies or ecotypes (Moritz et al. 2005). As a result of gene flow and direct replacement over longer distances (Peer 1957; Jensen and Pedersen 2005), native honey bees were almost extinct in many parts of Europe, such as in Germany (Maul and Hähnle 1994).

The Carpathian Basin Mountains represents one of the major mountain ranges of Europe, but still one of its least studied region. Several endemic taxa have been described from the Carpathian Mountains. The “hot spots” are considered to have a long‐term ecological stability, which cause the accumulation of the genetic information (Bálint et al. 2011). In this study, we analyzed the genetic diversity of native Carniolan Pannonian ecotype to determine the structure of the Carniolan Pannonian honey bee population in Hungary, paying special attention to detect introgression from neighboring subspecies. Accordingly, there is special importance to preserve this natural heritage of local populations, because it represents reservoirs of unique combinations of genes and adaptation to regional environmental factors (climate, vegetation, and prevailing disease) and requires adequate identification of the breeding material. The maladapted genes in the short term contribute to colony losses, and in long term, unsustainable (Meixner et al. 2014).

## Material and Methods

### Sampling and DNA extraction

**Figure 1:**
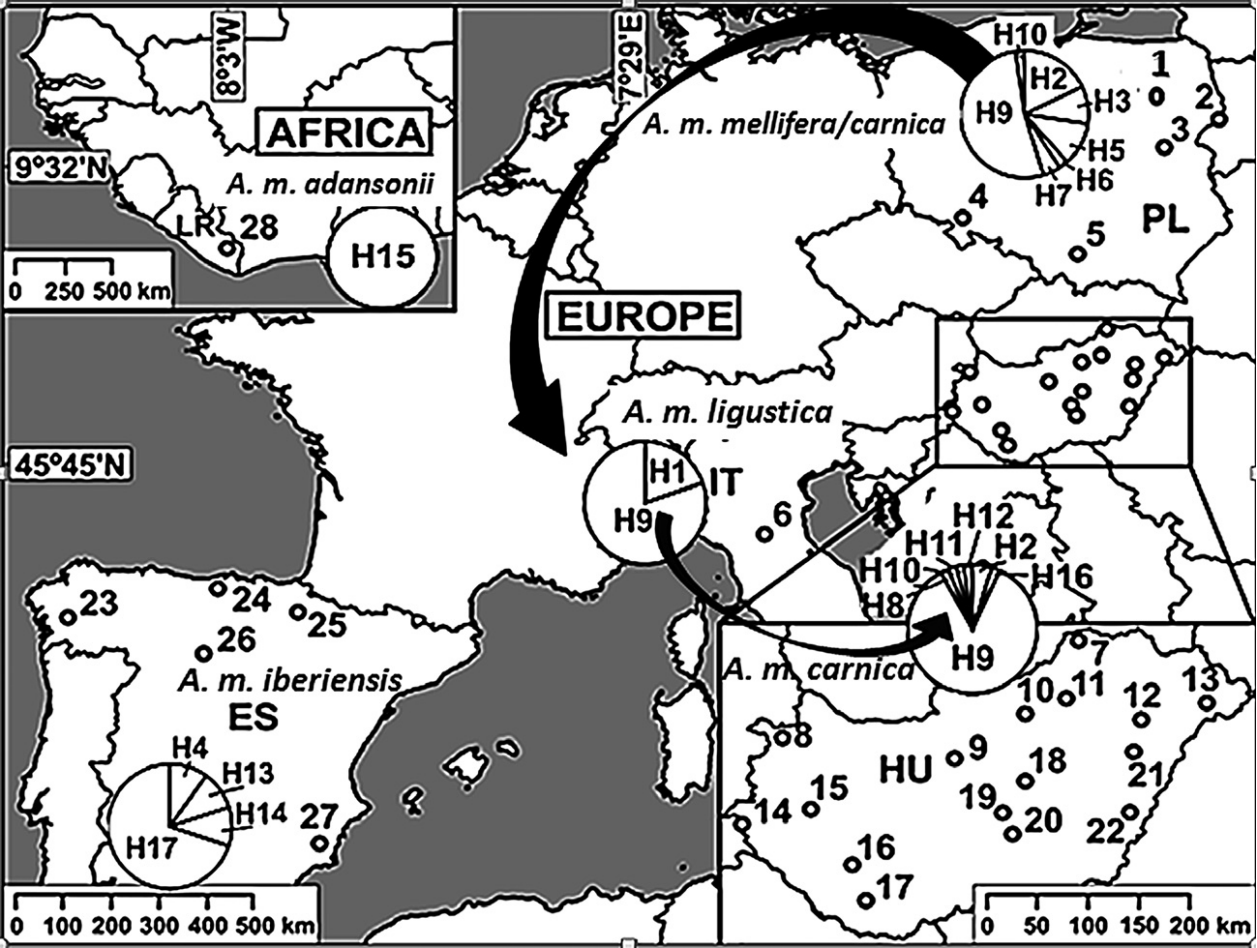
Maps of Europe and Africa that show the location where honey bee samples have been collected (1–28) and the distribution of haplotypes in the Hungarian, and the studied Italian, Polish, Spanish, and Liberian populations. Notations: HU – Hungary, IT – Italy, PL – Poland, ES – Spain, LR – Liberia1 – Augustów Forest, 2 – Bialowieza, 3 – Siedlce, 4 – Wroclaw, 5 – Krakow, 6 – Bologna, 7 – Perkupa, 8 – Fertőszentmiklós, 9 – Budapest, 10 – Gyöngyös, 11 – Cserépfalu, 12 – Hajdúvid, 13 – Nyírcsaholy, 14 – Kercaszomor, 15 – Bazsi, 16 – Kaposfüred, 17 – Dinnyeberki, 18 – Abony, 19 – Kecskemét, 20 – Kiskunfélegyháza, 21 – Hajdúszoboszló, 22 – Okány, 23 – Galícia, 24 – Cantabria, 25 – Navarra, 26 – Castilla Leon, 27 – Murcia, 28 – Jibloo.

Five‐ to seven‐day‐old worker pupae were sampled from 80 honey bee colonies in Hungary (*A. m. carnica*) at 16 different locations. Additional populations located in Italy (*A. m. ligustica*), Liberia (*A. m. adansonii*), Spain (*A. m. iberiensis*), Poland (*A. m. mellifera/carnica*), and the Buckfast line from Hungary were used for comparison (Fig. 1). Honey bees were individually placed in 1.5‐mL Eppendorf tubes containing 1 mL of 95% ethanol and kept at −20°C until they were processed in the laboratory.

One honey bee worker pupa per colony was used for mtDNA analysis (three individuals/locality) (*N* = 48) from Hungary.

Three honey bee pupae per colony were used for microsatellite analysis from Hungary (*N* = 240). All used samples are presented in Tables 1 and 2. Total DNA was extracted from worker pupae or adults according to Latorre et al. (1986).

**Table 1:** Summary of molecular diversity in mitochondrial DNA sequences of studied honeybee population

Populations	Subspecies	*N*	*N* hap	*Hd *± SD	*π *± SD
Hungary	*Apis mellifera carnica*	48	7	0.296 ± 0.060	0.0009 ± 0.001
Liberia	*Apis mellifera adansonii*	10	1	0.000 ± 0.000	0.000 ± 0.000
Spain	*Apis mellifera iberiensis*	10	4	0.533 ± 0.180	0.007 ± 0.004
Augustów	*Apis mellifera mellifera*	10	5	0.756 ± 0.130	0.007 ± 0.004
Krakow	*A. m. carnica*	10	3	0.600 ± 0.131	0.001 ± 0.001
Bialowieza	*A. m. mellifera*	9	2	0.389 ± 0.164	0.001 ± 0.001
Wroclaw	*A. m. carnica*	5	2	0.356 ± 0.159	0.004 ± 0.003
Siedlce	*A. m. mellifera*	10	2	0.400 ± 0.237	0.001 ± 0.001
Hungary	Buckfast line	10	1	0.000 ± 0.000	0.000 ± 0.000
Italy	*Apis mellifera ligustica*	10	2	0.356 ± 0.159	0.004 ± 0.003
Total		132	17	0.525 ± 0.045	0.004 ± 0.000

Number of individuals studied (*N*), number of haplotypes (*N* hap), haplotype (*Hd*), and nucleotide (*π*) diversity with standard deviation (SD).

**Table 2:** Multilocus microsatellite variation in the Hungarian and references honeybee populations

Populations	Subspecies	*N*	*n *± SD	*Ap *± SD	*Ho *± SD	*He *± SD	*Fis*
Hungary	*Apis mellifera carnica*	233	14.3 ± 6.2	3.667 ± 0.764	0.896 ± 0.224	0.657 ± 0.157	−0.366***
Liberia	*Apis mellifera adansonii*	15	8.1 ± 3.0	2.111 ± 0.588	0.985 ± 0.029	0.846 ± 0.048	−0.171***
Spain	*Apis mellifera iberiensis*	9	5.2 ± 2.7	0.444 ± 0.242	0.816 ± 0.307	0.644 ± 0.291	−0.154***
Augustów	*Apis mellifera mellifera*	15	6.4 ± 3.8	0.000 ± 0.000	0.881 ± 0.237	0.712 ± 0.151	−0.247***
Krakow	*A. m. carnica*	15	6.8 ± 3.2	0.111 ± 0.111	0.903 ± 0.200	0.734 ± 0.143	−0.238***
Bialowieza	*A. m. mellifera*	15	7.4 ± 2.6	0.222 ± 0.147	0.822 ± 0.270	0.747 ± 0.129	−0.104***
Wroclaw	*A. m. carnica*	6	3.7 ± 1.2	0.000 ± 0.000	0.907 ± 0.188	0.709 ± 0.103	−0.316ns
Siedlce	*A. m. mellifera*	15	6.1 ± 2.2	0.111 ± 0.111	0.888 ± 0.309	0.756 ± 0.098	−0.180***
Hungary	Buckfast line	10	4.3 ± 1.2	0.222 ± 0.222	0.843 ± 0.296	0.644 ± 0.217	−0.333***
Italy	*Apis mellifera ligustica*	15	5.3 ± 2.7	0.222 ± 0.222	0.911 ± 0.266	0.634 ± 0.190	−0.460***

Number of individuals studied (*N*), mean number of alleles per locus (*n*), frequency of private alleles (*Ap*), observed (*Ho*) and expected (*He*) heterozygosity with standard deviation (SD), and *Fis* value in all loci.

ns, not significant, ***P <* *0.001.

### Mitochondrial DNA analysis

The cox1 intergenic region was PCR‐amplified with the newly designed (due to fail in PCR amplification with the commonly used primers) forward (5′‐CTGATATAGCATTCCCCCGAATA‐3′) and reverse (5′‐AGAATTGGATCTCCACGTCCTA‐3′) primers. These primers were designed from 2056 to 2401 nucleotide position detected in the *A. m. ligustica* complete mitochondrial genome (Acc. no.: L06178.1) (Crozier and Crozier 1993). The 10 *μ*L reaction mix consisted of 1 *μ*mol/L of each primer, 0.2 mmol/L of PCR nucleotide mix (Fermentas, Lithuania), 3 mmol/L MgCl_2_ (Applied Biosystem), 10× reaction buffer (Applied Biosystem, Waltham, MA), 0.75 U Taq polymerase (Applied Biosystem), and 20 ng/*μ*L of template. The amplification cycle consisted of an initial denaturation step of 10 min at 95°C, followed by 35 cycles of 15 sec at 95°C, 30 sec at 63°C, and 30 sec at 73°C, followed by a final extension step of 25 min at 73°C. PCR products were purified using a Clean‐Up DNA fragment purification kit (A&A Biotechnology, Poland) and sequenced by the Eurofins MWG Operon Company (Ebersberg, Germany).

Each sequence obtained was manually checked and aligned with the published sequences for comparison using ClustalX program (Thompson et al. 1997). Haplotype determination and diversity index numbers were calculated using DnaSP version 5.10 software (Librando and Rozas 2009). A neighbor‐joining phylogenetic tree of all the haplotypes was reconstructed using the Jukes‐Cantor model and 10.000 bootstrap replicates using MEGA version 6.0 software (Tamura et al. 2013). The best suitable nucleotide substitution model was selected using the jModelTest 0.1.1 program (Posada 2009). In the course of the edited phylogenetic tree, we have chosen *Apis cerana* as an out‐group (Acc. no.: DQ020237.1) (Tan et al. 2011). The haplotype network analysis was carried out using a median‐joining algorithm and the Network version 4.61 software package (http://www.fluxus-engineering.com).

### Microsatellite analysis

Nine polymorphic microsatellite loci A7, A113, A107, A28, A88, A14, A35, A(B)24 (Estoup et al. 1995), and A43 (Garnery et al. 1998) were screened. The 25 *μ*L reactions contained 1 *μ*mol/L of each primer, 0.2 mmol/L of PCR dNTPs (Fermentas, Lithuania), 4.3 mmol/L MgCl_2_ (Promega, Fitchburg, WI), 5× reaction buffer (Promega, Fitchburg, WI), 0.8 U Taq polymerase (Promega, Fitchburg, WI), and 20 ng/*μ*L of extracted DNA. The amplification cycle consisted of an initial denaturation step of 2 min at 94°C, followed by 35 cycles of 30 sec at 94°C, 30 sec at 55°C (A (B) 24, A43), 56°C (A28, A88), 57°C (A35), 58°C (A107, A14, A7), 60°C (A113), and 30 sec at 72°C, followed by a final extension step of 10 min at 72°C.

Alleles were subsequently scored using PeakScanner version 1.0 software (Applied Biosystem). Population genetic parameters were calculated with GenAlEx 6.4 (Peakall and Smouse 2006) and Arlequin version 3.1 software (Excoffier et al. 2005). An exact test for genetic differentiation between populations using estimates of *Fst* was calculated using the FSTAT version 2.9.3. (Goudet 2001). The estimates of Nei's corrected standard genetic distance (*Ds*) (Nei 1978) were calculated with the PopGene package version 1.32 (Yeh et al. 1999).

Principal coordinate analysis (PCoA) and assignment test (Paetkau et al. 1995) were also performed using GenAlEx version 6.4 (Peakall and Smouse 2006). The individual genetic distances were calculated to find and plot the relationships between the individuals belonging to the different populations.

A clustering method was used for inferring population structure with STRUCTURE version 2.3.3. (Pritchard et al. 2000) software. This method estimated the posterior probability for a given number of *K* genetic populations, and an admixture model assuming correlated allele frequencies was used. In this study, the results were based on the simulations of 80.000 burn‐in steps and 1.000.000 MCMC (Markov chain Monte Carlo algorithm) iterations. Ten runs for each *K* value (2 ≤ *K* ≤ 10) were used, and the number of populations was reasoned from the value of ∆*K* as described in Evanno et al. (2005).

## Results

### Mitochondrial DNA

The sequence dataset sized 345 base pairs with 329 conserved and 15 variable positions from 180 *A. mellifera* individuals. The resulting sequences were compared to the reference sequence (Acc. no.: L06178.1, *Apis mellifera ligustica* complete mitochondrial genome). In 11 cases, the nucleotide exchanges were transitions, while in three cases, transversions (C/A, T/A, A/T), and in one case either a transition (G/A) or a transversion (G/C) took place in position 2169.

Seventeen different haplotypes (H1–17) were detected (GenBank accession numbers: under submission). Seven haplotypes have been characterized in the Hungarian population (H2, H8, H9 =  H16, H10, H11, and H12). The H9 is at high frequency in central European localities and is increasing in frequency toward the south. The ratio of H9 at Bialowieza (77.5%), Siedlce (80%), and Krakow (60%) line is relatively high, but from Augustów Forest (10%) to Wroclaw (20%) line is very low in Poland. The H8, H11, and H12 in Hungary; H4, H13, and H14 in Spain; and H5, H6, and H7 in Poland were detected at first. H15 was found only in Liberia (Fig. 1). H1, H10, and H17 were already published (corresponding to Ligus8, Carni3, Sicul2, respectively). In addition, the five more haplotypes of the cox1 segment are available in the NCBI GenBank database. H18, H19, and H20 correspond to Anato2, Iberi2, and Melli4 (Özdil and Ilhan 2012).

Haplotype diversity and nucleotide diversity values are presented in Table 1. The overall haplotype diversity and nucleotide diversity were 0.525 and 0.004, respectively. Haplotype diversity in the Hungarian population was low. The highest value of haplotype and nucleotide diversity was observed in the Augustów Forest population in Poland.

**Figure 2:**
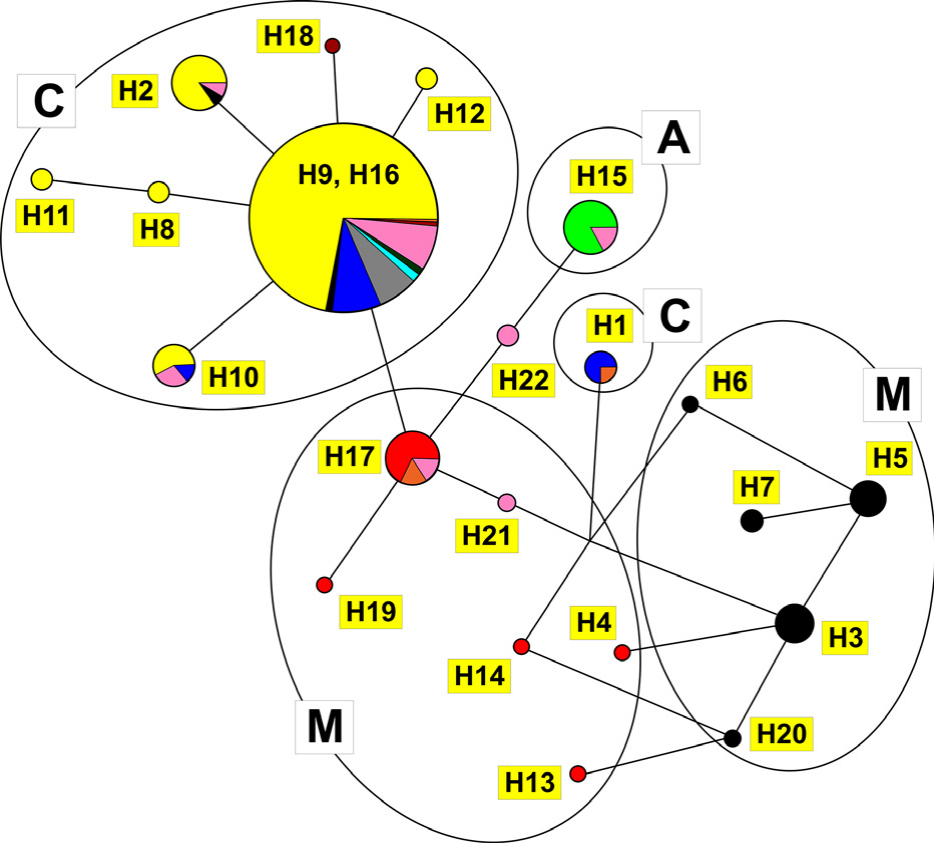
Median‐joining network analyses among the studied sequences. The size of the circles is proportional to the number of the haplotype individuals, and the different colors represent different subspecies. There are two mutations between H9 and H18, and among all other haplotype, only one. Color code: yellow – *A. m. carnica*, dark blue – *A. m. ligustica*, black – *A. m. mellifera*, gray – Buckfast line, light green – *A. m. adansonii*, red – *A. m. iberica*, orange – *A. m. sicula*, violet – *A. m. anatolica*, light blue – *A. m. caucasica*, dark green – *A. m. adami*, pink – sequence appearing in the NCBI GeneBank without the exact naming of the species.

Relationship among detected haplotypes was determined using median‐joining network. Haplotypes of the Carniolan subspecies were clustered together. The most common haplotype H9 was present in 1.3% of *A. m. mellifera*, 8.3% of *A. m. ligustica*, and 7.05% of Buckfast individuals. In addition to this, the more common haplotype in *A. m. carnica* (H2) was also found in the black honey bee (Fig. 2).

**Figure 3:**
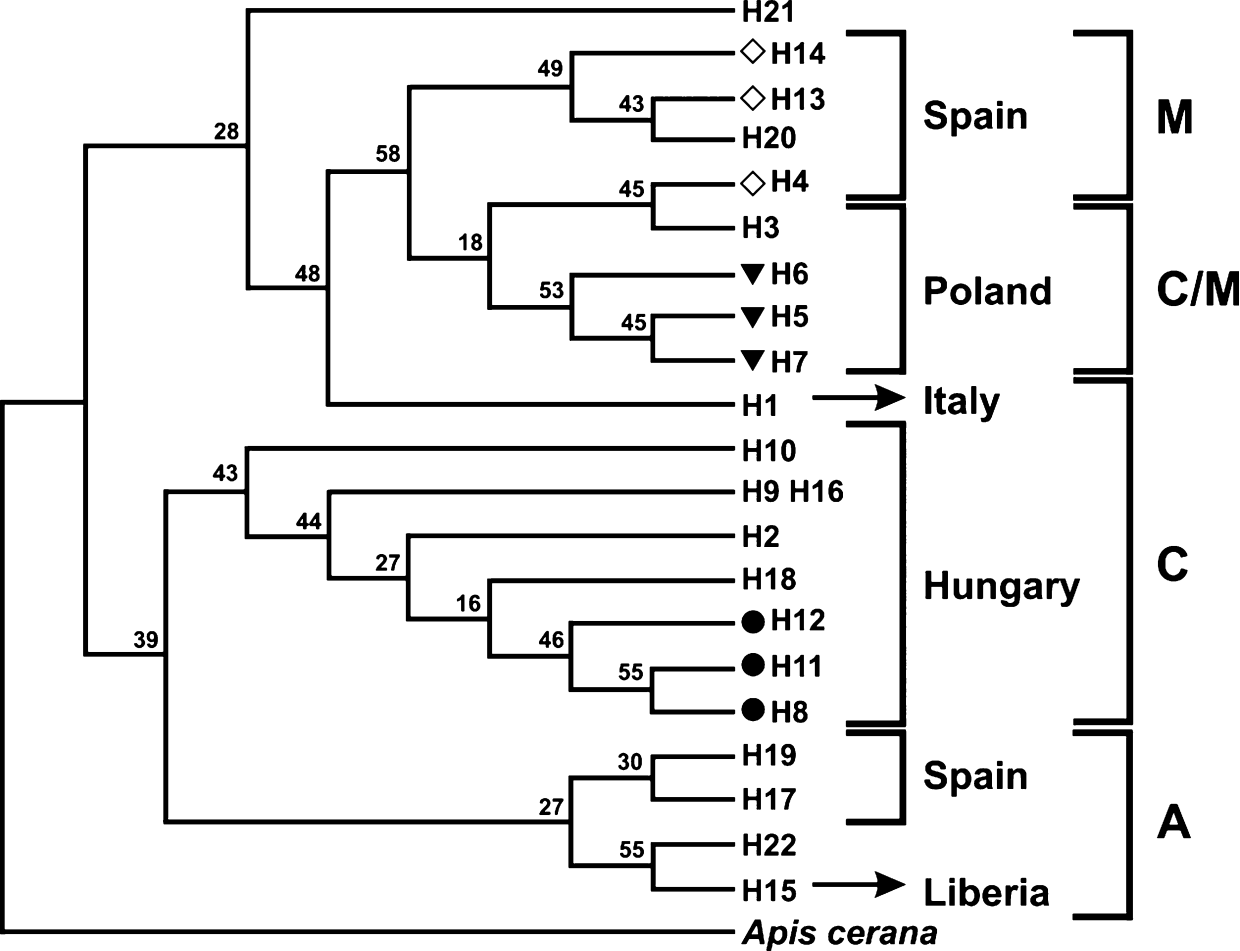
Neighbor‐joining phylogenetic tree performed by Jukes‐Cantor model, number of Bootstrap runnings: 10.000. (● – new haplotype from Hungary, ▼ – new haplotype from Poland, ♢ – new haplotype from Spain).

Haplotype sequences were aligned with those from 53 honey bee samples from GeneBank, and a neighbor‐joining phylogenetic tree was constructed showing nine novel haplotypes: Three new haplotypes marked with black circles (H8, H11, and H12) were detected in Hungary, three (H5, H6, and H7 labeled with black triangles) in Poland, and other three in Spain (H4, H13, and H14 marked with a blank rhombus). As expected, the Spanish and Liberian (H15) haplotypes were well differentiated from the Hungarian haplotypes (Fig. 3).

### Population structure based on microsatellite data

Overall parameters of the ten investigated populations and the *Fis* values are shown in Table 2. The average allele number varied between 3.7 (Wroclaw in Poland) and 14.3 (Hungary). The genetic diversity measured as expected heterozygosity (*He*), thus varied between 0.634 (Italy) and 0.846 (Liberia). Honey bee populations from Hungary deviated significantly from the Hardy–Weinberg equilibrium (*P* < 0.05). The *Fis* values of the ten groups varied between −0.104 (Bialowieza) and −0.460 (Italy), thus reflecting a heterozygote excess within all the populations with negative or close to zero values.

**Figure 4:**
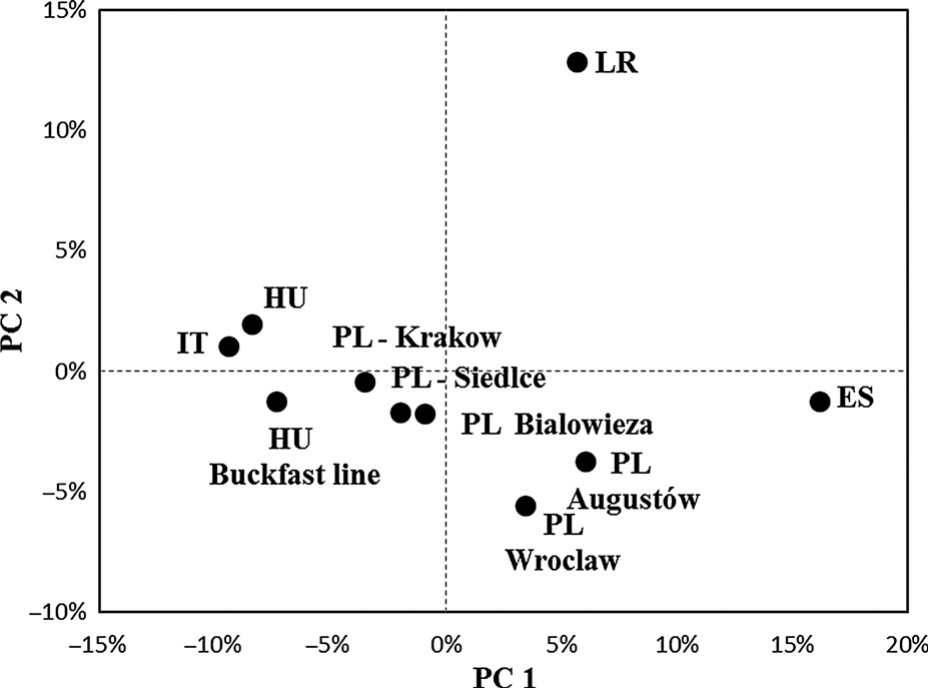
Principal coordinate analysis (PCoA) based on the distribution of *Apis mellifera* populations genetic distance. Notations: HU – Hungary, IT – Italy, PL – Poland, ES – Spain, LR – Liberia.

Principal coordinate analysis (PCoA) was performed to investigate population patterns based on the *Fst* genetic distance among individual samples. The results of principal coordinate analysis showed African (AF) and Spanish (SP) populations were well separated, while the Hungarian population (HU) appeared to be separated into two. On the other hand, Polish *A. m. mellifera* populations (Augustów Forest and Wroclaw) clustered separately from *A. m. carnica* subspecies from Krakow, Bialowieza, and Siedlce (Fig. 4).

**Figure 5:**
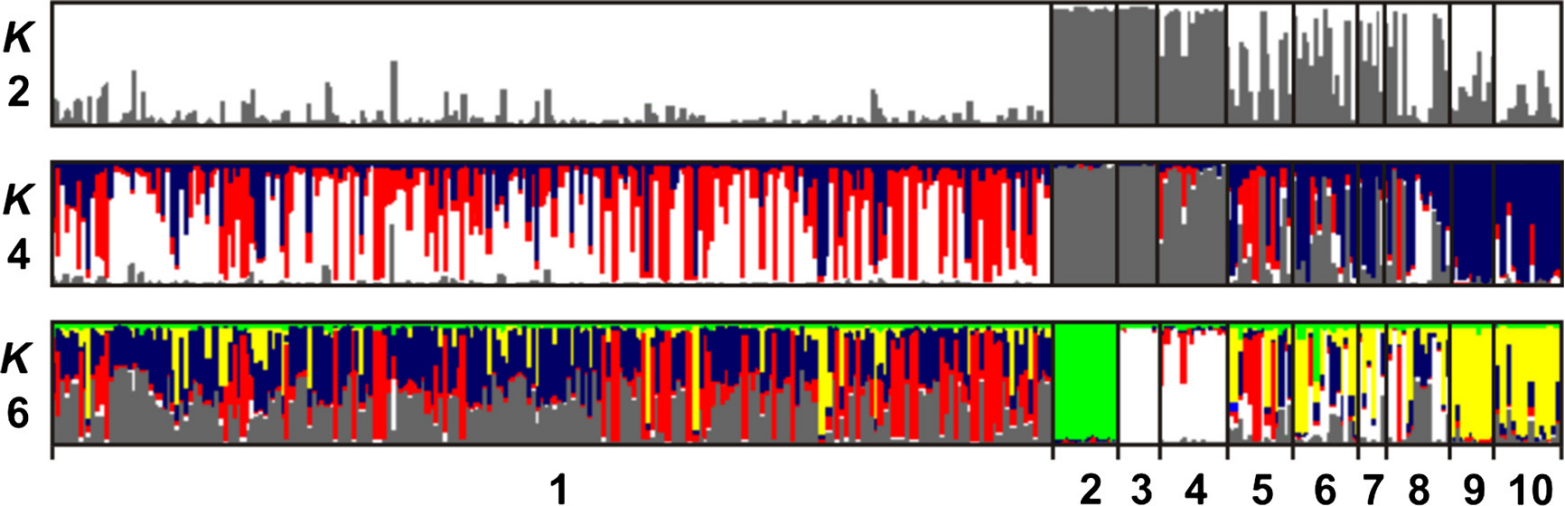
Cluster analysis of the studied honey bee populations (*K* = number of groups). Notations: 1 – Hungary, 2 – Liberia, 3 – Spain, 4 – Poland‐Augustów, 5 – Poland‐Krakow, 6 – Poland‐Bialowieza, 7 – Poland‐Wroclaw, 8 – Poland‐Siedlce, 9 – Buckfast line, 10 – Italy.

Cluster analysis of the honey bee populations (Fig. 5) identified the Hungarian Carniolan subspecies separated from the Liberian, Spanish, and Polish Augustów Forest subspecies and grouped into two populations (*K* = 2). This is supported by the *A. m. iberica* (Spain) and *A. m. mellifera* (Augustów) subspecies belong to the common M lineage. When the model assumed four populations (*K* = 4), the clustering revealed Buckfast and Italian populations separated from the other stock. The clustering together with the aforementioned two populations on the phylogenetic tree is also visible. Finally, *K* = 6 alignment the *A. m. adansonii* (Liberia) subspecies separation was seen by the *A. m. iberica* and *A. m. mellifera* (Poland‐Augustów) subspecies. The results showed the best *K* value after Evano correction (*K* = 2) (not shown).

### Population relationship and phylogeny

Similar results have been shown in the case of both the pairwise *Fst* values and the Nei's corrected standard genetic divergence (*Ds*) among the ten groups. Multilocus *Fst* values varied between −0.001 (Bialowieza and Wroclaw) and 0.291 (Hungary and Spain). Not surprisingly, the Liberian (0.216) and Spanish (0.291) populations were significantly distinct from the Hungarian population. Honey bees from Poland, which lie in the natural distribution range of *A. m. mellifera*, showed differentiated admixture of Carniolan bees. According to the examined microsatellite markers, the Polish Augustów Forest (0.167) and Wroclaw (0.136) populations showed strong divergence values from the Hungarian populations, stronger than the Bialowieza (0.084), Krakow (0.029), and Siedlce (0.045) populations. A genetic barrier has been identified among these populations, which is validated by the results of the PCoA at the population level (Fig. 4) and also the results of the mtDNA study. The divergence value of the black bee (Augustów Forest, Wroclaw) from our domestic Carniolan bee confirms that the *A. m. mellifera* subspecies was actually dominant in some parts of Poland.

Assignment tests to determine the breed of origin of individuals were performed. Only one individual from Wroclaw could be assigned to the *A. m. mellifera* subspecies. It was established that the presence of the black honey bee in Hungary was negligible. In addition to this, five individuals resembling *A. m. ligustica* and three belonging to the Buckfast hybrid were identified in the Hungarian stock. The results showed that the Polish population was widely heterogeneous, with some populations identified as *A. m. mellifera* and others as *A. m. carnica*. The majority of individuals of the eastern apiaries of Poland have not faded into the Hungarian population, which presumably is due to the differences within the subspecies (Table 3). Based on microsatellite variation, 93.6% of Hungarian samples were correctly allocated to their declared subspecies, while 6.4% were assigned to a different subspecies: Buckfast line (1.7%), the Italian bee (*A. m. ligustica*) (2.5%), and the black bee from Poland (*A. m. mellifera*) (2.2%). We confirmed that the Liberian individuals belong to the *A. m. adansonii* subspecies.

**Table 3:** Assignment test, results of the study at individual level

Individual	HU	AF	SP	AU	KR	BW	SI	WR	BU	IT	Number of studied bees (*n*)	Number of bees incorrectly assigned
Hungary	201	0	0	0	8	4	14	1	3	5	236	35
Liberia	0	15	0	0	0	0	0	0	0	0	15	0
Spain	0	0	10	0	0	0	0	0	0	0	10	0
Augustów	0	0	1	11	2	0	1	0	0	0	15	4
Krakow	3	0	0	1	5	2	4	0	0	0	15	10
Bialowieza	0	0	0	2	2	3	5	1	1	1	15	12
Siedlce	4	0	0	1	1	2	2	2	2	1	15	13
Wroclaw	0	0	0	0	0	0	1	5	0	0	6	1
Buckfast	0	0	0	0	0	0	2	0	8	0	10	2
Italy	3	0	0	0	0	0	1	0	0	11	15	4

HU, Hungary; AF, Liberia; SP, Spain; AU, Poland/Augustów; KR, Poland/Krakow; BW, Poland/Bialowieza; SI, Poland/Siedlce; WR, Poland/Wroclaw; BU, Buckfast line; IT, Italy.

## Discussion

In Central Europe, the haplotype 9 was the most frequent haplotype and its frequency decreased to the north. With the help of mtDNA and nine polymorph microsatellite markers, we proved that the Hungarian honey bee populations are nearly homogeneous. We identified heterozygosity in the domestic Carniolan Pannonian bees; therefore, inbreeding is not typical. In conclusion, the apicultural practices in the Hungarian honey bee colonies were appropriate for the conservation of indigenous honey bees. The results of this research provide new knowledge about genetic variability and useful information for conservation proposes by developing and supporting breeding programs. As it is well known, the health of honey bee colonies cannot be understood without considering the genetic diversity, and the locally adapted bees survived better than introduced bees.

The Hungarian population has been isolated geographically by the Carpathians Mountains northward, probably giving rise to the endemic honey bee population during the last glaciation (Coroian et al. 2014). The human activity and the absence of any southwest natural barrier could have caused slight introgression – what has already been recognized in phenotypes by the beekeepers – with other subspecies mainly from Italy (*A. m. ligustica*). Migratory beekeeping might explain weak introgression, very high excess of heterozygotes, and other rare haplo‐ and genotypes detected in the Hungarian population. In spite of this relative homogeneity, populations have developed one ecotype that showed relatively high heterozygosity and can survive in our climates, all features that are indicative of high evolutionary potential for local adaptation.

Dall'Olio et al. (2007) described the unique genetic characteristics of *A. m. ligustica* subspecies but did not found specific ecotypes within the local honey bee populations. In the present study, the H1 appeared only in the Italian population based on ten samples. The haplotype and nucleotide diversity of the investigated populations showed either average or above‐average rates, although there were significant differences in the number of samples in Hungarian and surrounding regions. According to Cánovas et al. (2007, 2011), the North African honey bees (A lineage) have colonized southwest of Europe (M lineage) and there was hybridization between lineages. Our results confirm this finding because on the neighbor‐joining phylogenetic tree, the H4, H13, H14 from Spain and H20 (Melli4) were closely related with the M lineage, and the H17 and H19 (Iberi2) also from Spain had closer connection with the A lineage (Fig. 3).

Microsatellite analysis is a suitable diagnostic method for confirming the origin of subspecies (Meixner et al. 2013). Using these markers, we have found that Hungarian honey bees are characterized by the genetic diversity levels that suggest low rates of inbreeding. The significant differences between observed and expected heterozygosity and diversity level could result by nonrandom mating, from beekeepers purchasing queens from breeders and features the intensive migratory movements within the country, what emphasized the importance to prevent the loss of this genetic diversity and to preserve ecotypes (De la Rúa et al. 2009). Our *A. m. carnica pannonica* ecotype is so valuable for world biodiversity. In comparison with the Italian populations (Dall'Olio et al. 2007), both Hungarian honey bees and *A. m. mellifera* used in this study as a reference showed lower heterozygosity values (0.470 and 0.375, respectively).

In the Structure, two different clusters were detected; thus, the Hungarian population was classified as nearly pure *A. m. carnica* (*K* = 2 was selected as the optimal populations). The population structure and genetic diversity of native Carniolan subspecies in Slovakia (He = 0.705) (Dusan et al. 2013), Poland (He = 0.734) (Stanimila et al. 2015), and Hungary (He = 0.657) were similar and showed high heterozygosity values and relatively high selection potential. In addition, the observed heterozygosity values of *A. m. carnica* population (Ho = 0.896) in Hungary were also at a similar level as the African Guinean (*A. m. adansonii*) population (Ho = 0.861) (Franck et al. 1998). More recent genetic studies (Franck et al. 1998; De la Rúa et al. 2007) implied that there is high genetic variability of African honey bee populations (0.756–0.896) (Franck et al. 2001). Our results showed similar high diversity values in Carniolan subspecies in Hungary (0.896). It shows this part of Europe (Carpathian Basin) is an important present refuge. This finding is concordant with other studies that have found high diversity to marbled white butterfly (*Melanargia galathea*) and wild bees (*Apoidae*) (Schmitt et al. 2006; Sárospataki et al. 2009) and refugia of other insects, such as *Isophya* species and red‐tailed bumblebee (*Bombus lapidarius*) (Bauer and Kenyeres 2006; Lecocq et al. 2013) in Carpathian Basin. Observed levels of genetic variability and heterozygosity were relatively high in continental Europe and among *Bombus terrestris* commercial populations (Moreira et al. 2015).

If the microsatellite average allele number had been considered in a recent study, we received higher values (14.3) than Dall'Olio et al. (2007) for the reference *A. m. carnica* (6.6) population. Our data comparison differs in the results of western European populations because the number of heterozygosity of microsatellite alleles was reduced (De la Rúa et al. 2003), such as in Spain (Estoup et al. 1995). In addition, there are lower heterozygosity values (0.647) in the Croatian *A. m. carnica* population, and Muñoz et al. (2009) could account for two well‐separated subpopulations in contrast to the recent results.

At the population level, pairwise *Fst* values and Nei's corrected standard genetic divergence values (*Ds*) revealed the strong differentiation among Liberian, Spanish, and Hungarian populations. This suggested that the geographic distance was an impediment to the gene flow among colonies. The Hungarian Carniolan honey bee population showed slight distance values from the black honey bee and Italian subspecies. Dall'Olio et al. (2007) demonstrated the *A. m. carnica* and *A. m. ligustica* introgression in the northern natural hybridization zone in Italy. In the present study, the assignment test predicted that 20% of the Italian bees and 12.1% of the Polish bees could be assigned to the same genetic cluster as Hungarian bees.

The European black bee has been present to Poland and northern Ukraine and hybridizes with subspecies of the C lineage (Meixner et al. 2007), such as the *A. m. carnica* subspecies from Balkan countries and *A. m. macedonica* from southwest of Europe which are more frequent in regions with mean temperatures above 9°C (Coroian et al. 2014). Recently, considerable amount of Buckfast alleles appeared in the Polish population (Francis et al. 2014). Eastern part of Poland that showed supposedly Carniolan aspect was separated well from the Hungarian Carniolan Pannonian populations. It follows that separation may have ensued inside a subspecies presumably. Recent study found that climate is the main factor which drives to the distribution of honey bee differences rather than geography barriers, like mountains (Carpathian) (Coroian et al. 2014). Our results of the genetic divergence values were equally confirmed by the assignment test and the population‐level principal coordinate analysis. However, the calculations of Structure software concluded that only the population from Augustów Forest was differentiated from the Hungarian populations.

The assignment test also revealed the presence of non‐*A. m. carnica* alleles in the studied populations. Moreover, the Hungarian population was basically homogeneous (93.6%), although a small‐scale gene flow was observed. It was established that the presence of the black honey bee in Hungary was negligible. De la Rúa et al. (2009) mentioned that native nonhybridized *A. m. carnica* populations still exist in Croatia, Serbia, and Slovenia. Because of the small‐scale indigenous gene affect, the Hungarian populations also reckon among these countries.

Based on the results, the Polish population was considerably heterogeneous. Oleksa et al. (2011) confirmed this result by describing that approximately 10–30% of the nuclear gene pool and 3–50% of mitochondria revealed the presence of hybrids in the studied Polish populations from northeast of Poland. In other population from northern Poland, Oleksa and Tofilski (2015) based on microsatellites classified 57.9% of workers as pure black bees, 12.1% as pure Carniolan bees, and 30.0% as hybrids. The reasons presumably result from the importation of alien honey bee queens and the natural hybridization, which appeared also in Hungary, but the data currently suggest a slight measure of gene flow. Moreover, Francis et al. (2014) described the beekeepers used hybrids between *A. m. carnica* and *A. m. caucasica* and widely propagated them in Poland. The native honey bee populations, such as *A. m. mellifera*, has been replaced by *A. m. carnica* in several regions of Central Europe, which may be ascribed to insufficient mating control (Kotthoff et al. 2013).

We suggest using instrumental insemination with sufficiently examined sperm donors throughout Hungary, which is important in preventing introgression and hybridization. Furthermore, it is important to assess the prevalence of the H9 from neighboring regions. We hope that our results may provide additional important novel evidences for the conservation of the native Carniolan honey bee populations in Central and eastern Europe. Data about mitochondrial and microsatellite DNA polymorphism in native Hungarian honey bees were reported here for the first time, and the two molecular tools showed near‐concordant result.

## Conflict of Interest

None declared.

## Supporting information


**Figure S1.** Neighbour‐joining tree using Nei genetic distance.
