# Supplementary material for: Population structure of honey bees in the Carpathian Basin (Hungary) confirms introgression from surrounding subspecies
Source: Ecol Evol. 2015 Nov 4;5(23):5456–67. doi: 10.1002/ece3.1781 (PMC4813114; doi:10.1002/ece3.1781)
Supplement: Supplementary file 1 — Figure S1. Neighbour‐joining tree using Nei genetic distance. [file ECE3-5-5456-s001.docx]

**Supplementary Information**


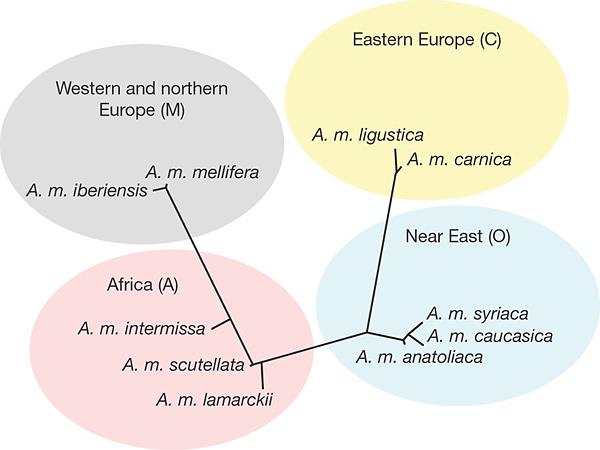


**Fig. S1:** Neighbour-joining tree using Nei genetic distance. Ten geographical subspecies (*N* = 9–21 individuals each) can be partitioned into four regional groups. Branches separating regional groups are supported by 100% bootstrap (The Honeybee Genome Sequencing Consortium, 2006).
